# Author Correction: Chromosome-level genome assembly of *Bactrocera dorsalis* reveals its adaptation and invasion mechanisms

**DOI:** 10.1038/s42003-022-03456-z

**Published:** 2022-05-17

**Authors:** Fan Jiang, Liang Liang, Jing Wang, Shuifang Zhu

**Affiliations:** 1 Chinese Academy of Inspection and Quarantine, Beijing, 100176 China; 2 Academy of Agricultural Planning and Engineering, MARA, Beijing, 100125 China; 3 Biomarker Technologies Corporation, Beijing, 101300 China; 4 Sanya Research Institute of Chinese Academy of Tropical Agricultural Sciences, Hainan, 572025 China

**Keywords:** Genome, Invasive species, Molecular evolution

Correction to: *Communications Biology* 10.1038/s42003-021-02966-6, published online 11 January 2022.

The original version of this Article was missing data accessions (SRR19134783 and SRR19134809) in the Data Availability statement.

“All genome sequence data are available at the GenBank under the Accession number JABETM000000000. Raw sequence data are available at the NCBI SRA site with the accession numbers SRR15444039 and SRR15254972.”

This error has been corrected in the HTML and PDF versions of the Article, as follows:

“All genome sequence data are available at the GenBank under the Accession number JABETM000000000. Raw sequence data are available at the NCBI SRA site with the accession numbers SRR15444039, SRR15254972, SRR19134783 and SRR19134809.”

