# Supplementary material for: Chromosome-level genome assembly of Bactrocera dorsalis reveals its adaptation and invasion mechanisms
Source: Commun Biol. 2022 Jan 11;5:25. doi: 10.1038/s42003-021-02966-6 (PMC8752857; doi:10.1038/s42003-021-02966-6)
Supplement: Supplementary file 2 — Supplementary Information [file 42003_2021_2966_MOESM2_ESM.pdf]

Supplementary Materials

Supplementary Tables

Supplementary Table 1. Statistics for the Hi-C and quality assessments for the Hi-C library

	Library 1	Library 2
Read Pairs Number	139,051,915	140,607,185
Base Number	41,664,686,002	42,131,648,670
GC Content (%)	38.33	38.05
%≥Q30	92.70	92.29
Total Read Pairs	139,051,915 (100%)	140,607,185 (100%)
Mapped Reads	173,562,864 (62.41%)	174,183,978 (61.94%)
Unique Mapped Read Pairs	49,548,526 (35.63%)	49,181,996 (34.98%)
Unique Paired Alignments	49,548,526 (100%)	49,181,996 (100%)
Valid Interaction Pairs	10,718,535 (21.63%)	10,702,134 (21.76%)
Dangling End Pairs	24,280,178 (49%)	24,167,705 (49.14%)
Re-ligation Pairs	475,746 (0.96%)	464,382 (0.94%)
Self-cycle Pairs	9,086,067 (18.34%)	9,068,006 (18.44%)
Dumped Pairs	4,988,000 (10.07%)	4,779,769 (9.72%)

Supplementary Table 2. Statistics for the Hi-C assembly of the *Bactrocera dorsalis* genome

Group	Sequence Number	Sequence Length (bp)
Lachesis Group0	274	111,840,857
Lachesis Group1	190	110,264,438
Lachesis Group2	313	99,045,911
Lachesis Group3	216	73,823,640
Lachesis Group4	195	55,676,428
Lachesis Group5	239	46,420,759
Total Sequences Clustered	1,427 (55.33%)	497,072,033 (92.35%)
Total Sequences Ordered and Oriented	860 (60.27%)	466,930,545 (93.94%)

Supplementary Table 3. Summary of the *Bactrocera dorsalis* genome assembly data based on PacBio Sequel and Hi-C data

Method	Contig number	Contig length (bp)	Contig N50 (bp)	Contig N90 (bp)	Contig max (bp)	GC content (%)
PacBio Sequel	2,403	538,097,872	1,059,842	128,381	8,111,928	36.80
Hi-C correction	2,579	538,241,858	862,733	109,166	7,710,970	36.79
Hi-C validation	2,379	542,036,191	1,122,451	117,280	8,109,558	36.79

Supplementary Table 4. Genome assembly and annotation summary for *Bactrocera dorsalis* and 13 other species

Species	Bdo	Bla	Bol	Zcu	Cca	Rze	Dme	Lcu	Mdo	Bmo	Sli	Dpl	Pxy	Lmi
Level	Chromosome	Scaffold	Scaffold	Scaffold	Scaffold	Scaffold	Chromosome	Scaffold	Scaffold	Chromosome	Chromosome	Chromosome	Scaffold	Contig
Size (Mb)	542.04	462.505	403.075	374.82	436.48	1113.96	137.577	378.27	750.4	429.019	438.956	248.627	393.46	5759.8
Number of contigs	2,379	30,469	48,618	43,002	3,243	139,583	2,442	11,857	104,054	727	13,636	10,791	38,757	1,397,492
Number of scaffolds	1,694	3,306	38,161	5,572	2,355	86,670	1,870	5,858	20,487	697	3,597	4,115	1,794	_
Contig N50 (Mb)	1.12	0.030	0.179	0.017	0.807	0.018	20.490	0.090	0.011	11.636	0.065	0.103	0.018	0.009
Scaffold N50 (Mb)	90.46	0.929	4.359	1.334	1.588	0.060	24.116	0.263	0.216	16.018	0.873	8.783	0.703	_
G + C (%)	36.79	37.0997	35.1998	37.2996	35.1997	37.3	42.0751	29.9	36.1	38.15	36.8924	32.1008	39.8	40.7
Number of genes	15,775	14,091	13,590	14,650	14,223	29,399	17,864	16,785	17,410	16,880	17,426	14,669	19,351	17,586

Abbreviations: Bdo (*Bactrocera dorsalis*), Bla (*Bactrocera latifrons*), Bol (*Bactrocera oleae*), Zcu (*Zeugodacus cucurbitae*), Cca (*Ceratitis capitata*), Rze (*Rhagoletis zephyria*), Dme (*Drosophila melanogaster*), Lcu (*Lucilia cuprina*), Mdo (*Musca domestica*), Bmo (*Bombyx mori*), Sli (*Spodoptera litura*), Dpl (*Danaus plexippus*), Pxy (*Plutella xylostella*), Lmi (*Locusta migratoria*). These data were obtained from NCBI Assembly (https://www.ncbi.nlm.nih.gov/assembly/) and Genome database (https://www.ncbi.nlm.nih.gov/genome).

Supplementary Table 5. CEGMA results of the integrity of the assembled genome

Species	Number of 458 CEGs present in the assembly	% of 458 CEGs present in assemblies	Number of 248 highly conserved CEGs present	% of 248 highly conserved CEGs present
Bactrocera dorsalis	457	99.78%	246	99.19%

CEGMA v2.5 database contains 458 conserved core genes of eukaryotes (CEGs). CEGMA v2.5 was used to evaluate the integrity of genome assembly. In the genome we assembled, 457 (99.78%) of 458 core genes were found by identity > 70% alignment. Among the 248 more conserved sequences in CEGMA v2.5, 246 (99.19%) could be found in the assembled genome.

Supplementary Table 6. BUSCO results of the integrity of the assembled genome

Species	Complete BUSCOs	Complete and single-copy BUSCOs	Complete and duplicated BUSCOs	Fragmented BUSCOs	Missing BUSCOs
Bactrocera dorsalis	2,714 (96.96%)	2,691 (96.14%)	23 (0.82%)	50 (1.79%)	35 (1.25%)

The Diptera database in BUSCO v2.0 contains 2799 conserved core genes in Diptera. We used BUSCO v2.0 software to evaluate the integrity of the assembled genome. Among the assembled genes, 2714 complete BUSCO genes were found, including 2691 single copy genes, 50 fragmented BUSCO genes, and 35 genes not found in Diptera library.

Supplementary Table 7. Summary of the functional annotation of protein-coding genes in the genome of *Bactrocera dorsalis* based on five databases

Database	Annotated number	Percentage (%)
GO Annotation	8,981	56.93
KEGG Annotation	5,639	35.75
KOG Annotation	9,607	60.90
TrEMBL Annotation	15,155	96.07
NR Annotation	15,296	96.96
All Annotated	15,382	97.51

Abbreviations: GO (Gene Ontology), KEGG (Kyoto Encyclopedia of Genes and Genomes), KOG (Eukaryotic Orthologous Groups), NR (Non-Redundant Protein Sequence Database).

Supplementary Table 8. Statistics for repeat elements in the genome of *Bactrocera dorsalis*

Types	Number	Length (bp)	Percentage (%)
Class I			
DIRS	2,417	4,624,928	0.85
LINE	4,198	9,045,339	1.67
LTR	16,107	25,428,120	4.69
LTR/Copia	1,276	1,920,926	0.35
LTR/Gypsy	17,313	29,049,175	5.36
PLE|LARD	14,148	7,072,638	1.3
SINE	239	57,040	0.01
TRIM	7,938	4,253,670	0.78
Unknown	1,022	128,518	0.02
Class II			
Crypton	242	16,817	0
Helitron	72,863	26,528,061	4.89
MITE	1,166	310,334	0.06
Maverick	1,532	128,305	0.02
TIR	305,451	178,000,404	32.84
Unknown	9,129	919,220	0.17
PotentialHostGene	485	238,529	0.04
SSR	137	21,149	0
Unknown	22,207	4,971,361	0.92
Total with overlap	477,870	292,714,534	54
Total without overlap	477870	250,364,057	46.19

Supplementary Table 9. Statistics for noncoding RNA genes, pseudogenes, and predicted motifs in the genome of *Bactrocera dorsalis*

Types	Number	Family
miRNA	59	44
rRNA	32	4
tRNA	493	24
snRNA	41	6
snoRNA	170	2
Pseudogene	1,393	
Motif	1,600	
Domain	28,659	

Supplementary Table 10. Duplicated genes and DDEs in *Bactrocera dorsalis* and 13 other insect species

Species	Tandemly Duplicated Gene		Segmental Duplicated Gene		DDEs			
	Numbers	Numbers/ All genes (%)	Numbers	Numbers/ All genes (%)	Numbers	Numbers/ All genes (%)	Numbers in TDGs	Numbers in SDGs
Bactrocera dorsalis	2186	13.86%	233	1.48%	121	0.77%	2	40
Bactrocera latifrons	477	3.78%	0	0.00%	67	0.53%	0	0
Bactrocera oleae	684	5.26%	0	0.00%	15	0.12%	1	0
Zeugodacus cucurbitae	245	2.07%	0	0.00%	67	0.53%	0	0
Ceratitis capitata	192	1.55%	10	0.08%	25	0.20%	0	0
Rhagoletis zephyria	149	0.92%	0	0.00%	402	1.58%	32	0
Drosophila melanogaster	1888	13.56%	70	0.50%	7	0.05%	0	0
Musca domestica	1208	8.19%	0	0.00%	40	0.27%	0	0
Lucilia cuprina	683	4.42%	49	0.32%	34	0.22%	0	0
Bombyx mori	2336	13.84%	320	1.90%	26	0.15%	0	0
Spodoptera litura	149	0.92%	30	0.19%	36	0.22%	0	19
Danaus plexippus	2460	16.26%	24	0.16%	8	0.05%	0	0
Plutella xylostella	567	3.16%	207	1.15%	67	0.37%	0	0
Locusta migratoria	1219	6.92%	0	0.00%	5	0.03%	0	0

Note: "TDGs” indicates Tandemly Duplicated Genes; “SDGs” indicates Segmental Duplicated Gene; “DDEs” indicates DDE superfamily endonuclease genes.

Supplementary Table 11. Information regarding gene family clustering in the 14 species used for comparative analyses

Item	Bdo	Bla	Bol	Zcu	Cca	Rze	Dme	Lcu	Mdo	Bmo	Sli	Dpl	Pxy	Lmi
Number of genes	15,775	12,609	13,003	11,825	12,398	25,452	13,576	15,438	14,754	16,815	16,122	15,123	17,966	17,363
Number of genes in orthogroups	13,705	12,047	12,117	11,235	11,576	19,114	10,975	13,245	12,849	14,099	13,679	11,441	14,810	9,387
Number of unassigned genes	2,070	562	886	590	822	6,338	2,601	2,193	1,905	2,716	2,443	3,682	3,156	7,976
Percentage of genes in orthogroups	86.9	95.5	93.2	95	93.4	75.1	80.8	85.8	87.1	83.8	84.8	75.7	82.4	54.1
Percentage of unassigned genes	13.1	4.5	6.8	5	6.6	24.9	19.2	14.2	12.9	16.2	15.2	24.3	17.6	45.9
Number of orthogroups containing species	11,448	10,620	10,575	9,763	10,175	12,147	8,920	10,600	9,968	10,037	10,723	9,987	10,126	6,607
Percentage of orthogroups containing species	46.6	43.2	43	39.7	41.4	49.4	36.3	43.1	40.6	40.8	43.6	40.6	41.2	26.9
Number of species-specific orthogroups	73	12	48	21	23	674	195	381	192	266	249	98	435	450
Number of genes in species-specific orthogroups	171	34	104	45	80	2,169	590	916	595	2,225	797	253	1,205	1,593
Percentage of genes in species-specific orthogroups	1.1	0.3	0.8	0.4	0.6	8.5	4.3	5.9	4	13.2	4.9	1.7	6.7	9.2

Abbreviations: Bdo (*Bactrocera dorsalis*), Bla (*Bactrocera latifrons*), Bol (*Bactrocera oleae*), Zcu (*Zeugodacus cucurbitae*), Cca (*Ceratitis capitata*), Rze (*Rhagoletis zephyria*), Dme (*Drosophila melanogaster*), Lcu (*Lucilia cuprina*), Mdo (*Musca domestica*), Bmo (*Bombyx mori*), Sli (*Spodoptera litura*), Dpl (*Danaus plexippus*), Pxy (*Plutella xylostella*), Lmi (*Locusta migratoria*). The raw data were obtained from the NCBI database and used for comparative analyses after filtering and removing redundant data.

Supplementary Table 12. Gene orthology comparison between *Bactrocera dorsalis* and 13 other insect species

Species	1:1:1	N:N:N	Orthoptera	Lepidoptera	Diptera	Tephritidae	*Bactrocera*	*Zeugodacus*	*Ceratitis*	*Rhagoletis*	SD	ND	Others
Bactrocera dorsalis	2250	783	0	0	1318	340	92	0	0	0	73	171	8839
Bactrocera latifrons	2291	670	0	0	1259	313	87	0	0	0	12	34	8072
Bactrocera oleae	2278	715	0	0	1283	322	89	0	0	0	48	104	7991
Zeugodacus cucurbitae	2305	670	0	0	1295	336	0	45	0	0	21	45	7206
Ceratitis capitata	2297	672	0	0	1253	322	0	0	80	0	23	80	7616
Rhagoletis zephyria	2338	1604	0	0	1452	365	0	0	0	2169	674	2169	8937
Drosophila melanogaster	2211	879	0	0	1312	0	0	0	0	0	195	590	6189
Musca domestica	2244	938	0	0	1544	0	0	0	0	0	192	595	7240
Lucilia cuprina	2285	718	0	0	1402	0	0	0	0	0	381	916	7683
Bombyx mori	2280	686	0	1558	0	0	0	0	0	0	266	2225	7235
Spodoptera litura	2250	833	0	1624	0	0	0	0	0	0	249	797	7938
Danaus plexippus	2243	758	0	1495	0	0	0	0	0	0	98	253	7353
Plutella xylostella	2084	1691	0	1801	0	0	0	0	0	0	435	1205	7155
Locusta migratoria	2186	1030	1593	0	0	0	0	0	0	0	450	1593	3621

Note: "1:1:1" indicates single-copy genes in all species; "N:N:N" indicates multi-copy genes in all species; "Orthoptera" indicates orthopteran insect-specific genes; "Lepidoptera" indicates lepidopteran insect-specific genes; "Diptera" indicates dipteran insect-specific genes; "Tephritidae" indicates tephritid insect-specific genes; "*Bactrocera*" indicates *Bactrocera*-specific genes; "*Zeugodacus*" indicates *Zeugodacus*-specific genes; "*Ceratitis*" indicates *Ceratitis*-specific genes; "*Rhagoletis*" indicates *Rhagoletis*-specific genes; "SD" indicates species-specific duplicated genes; "ND" indicates species-specific genes; "Others" indicates all other orthologous groups.

Supplementary Table 13. Manual annotations of detoxification related genes

	P450					GST
	CYP2	CYP3	CYP4	Mito	Total	
Bactrocera dorsalis	7	45	35	15	**102**	**34**
Bactrocera latifrons	9	49	39	22	**119**	**33**
Bactrocera oleae	8	44	28	18	**98**	**33**
Zeugodacus cucurbitae	7	43	39	21	**110**	**33**
Ceratitis capitata	7	51	23	17	**98**	**28**
Drosophila melanogaster	11	54	39	19	**123**	**36**
Locusta migratoria	8	40	22	7	**77**	**26**

Supplementary Table 14. Statistics for sequencing data for *Bactrocera dorsalis* genome assembly

Method	Insert size (bp)	Data (Gb)	Coverage (X)	Usage
Illumina HiSeq	270	29.37	56	Survey, correction
PacBio Sequel	20,000	31.45	57	De novo assembly
Hi-C technology	300-700	83.79	155	Chromosome-level assembly
Total	/	144.61	268	/

Supplementary Figures:

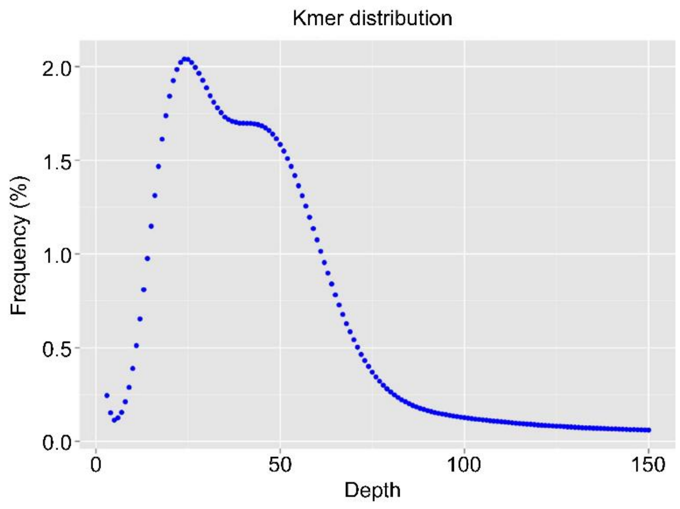

Supplementary Figure 1. Analysis of the genome size, heterozygosity, and duplication rate of the *Bactrocera dorsalis* genome. Illumina short-read sequencing data were used to count k-mers in DNA with k = 19. The estimated genome size of *Bactrocera dorsalis* was about 522.76 Mb. The rate of repeat sequences was about 41.46%. Heterozygosity was about 2.2%. The GC content was about 37.86%.

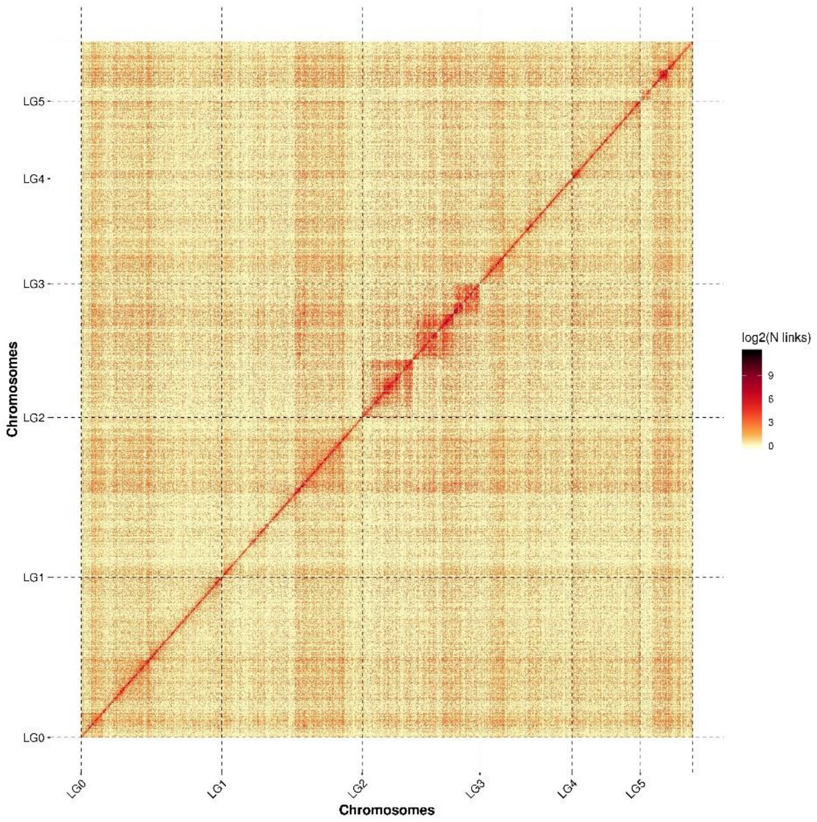

Supplementary Figure 2. Hi-C interaction matrix for *Bactrocera dorsalis* genome assembly using six clusters. LG0 to LG5 indicate six chromosomes in *B. dorsalis*. Strong interactions are indicated in dark red, and weak interactions are indicated in yellow.

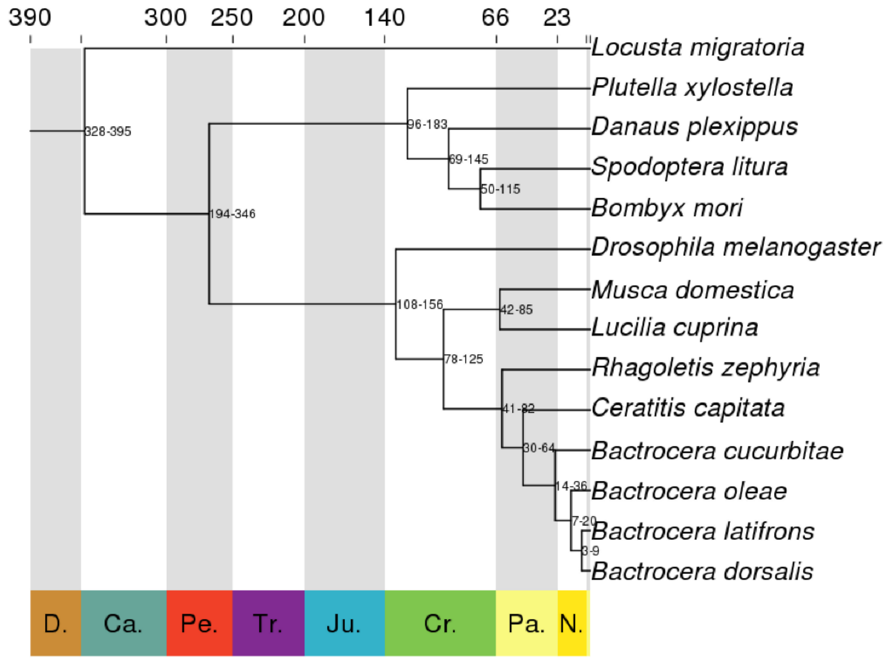

Supplementary Figure 3. Maximum-likelihood phylogenetic analysis for 14 species based on genomic data. The numbers next to the nodes are the estimated node ages in a million years. The phylogenetic relationship was estimated using a maximum likelihood analysis of a concatenation of 786 single-copy orthologous protein sequences, 1000 bootstrap replicates, and *Locusta migratoria* rooting. D, Ca, Pe, Tr, Ju, Cr, Pa, and N indicate Devonian, Carboniferous, Permian, Triassic, Jurassic, Cretaceous, Paleogene, and Neogene, respectively.

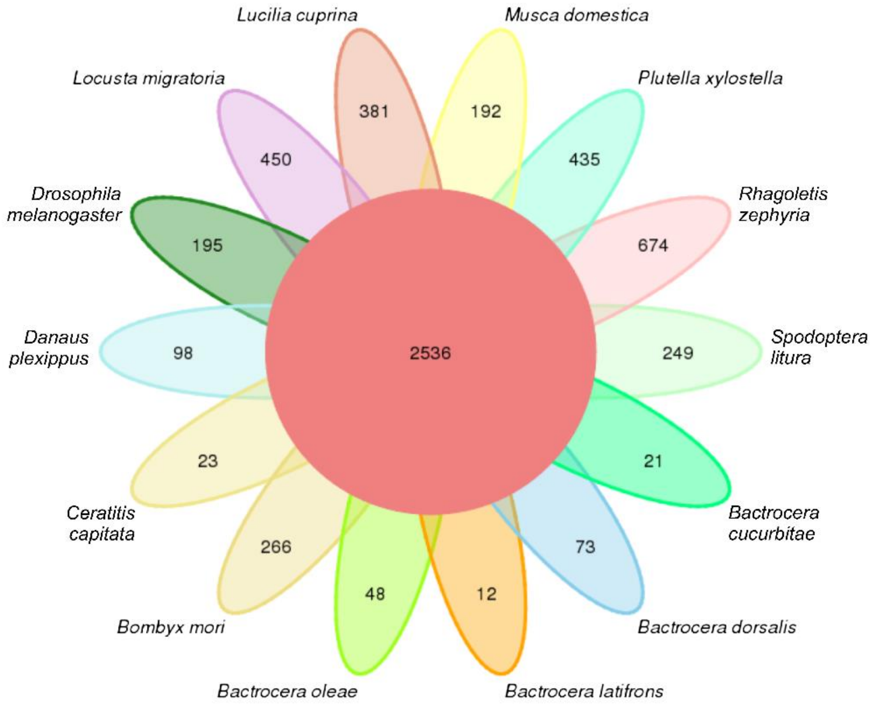

Supplementary Figure 4. Petal diagram of gene family clustering. The middle circle is the number of gene families common to all species, and on edge are the numbers of gene families unique to all component species.

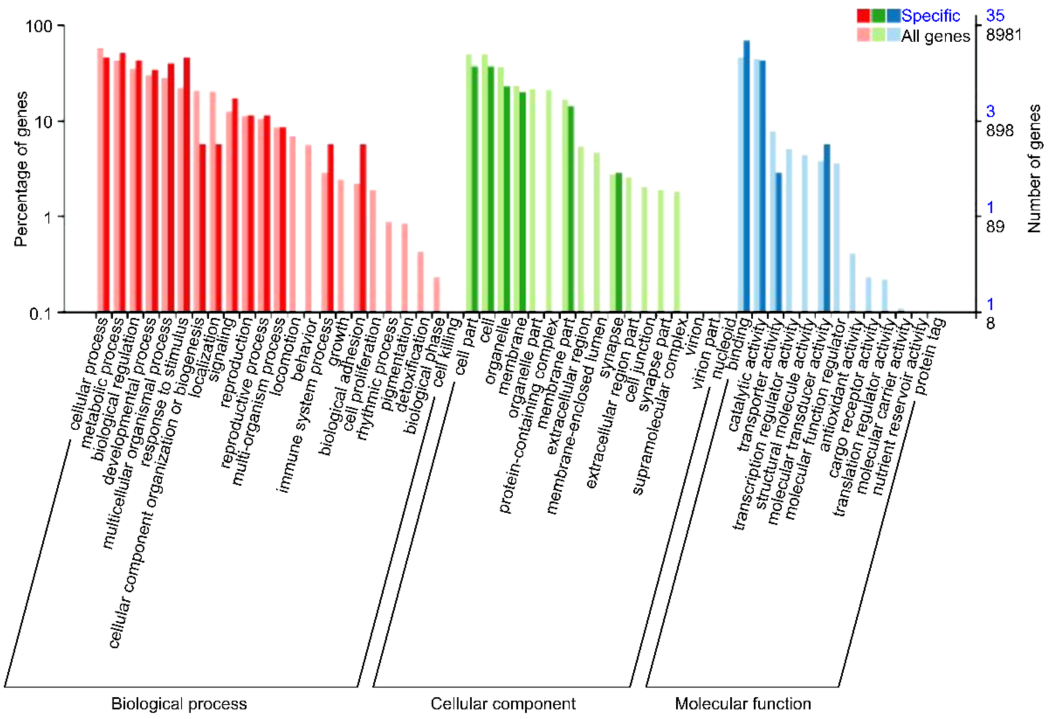

Supplementary Figure 5. All enriched GO terms for the gene families depicted in *Bactrocera dorsalis*-specific genes. Overrepresented GO terms were found for specific gene families in *Bactrocera dorsalis*. The x-axis presents the three GO terms biological process, cellular component, and molecular function. The y-axes in the left and right indicate the percentage of genes and number of genes, respectively. The red, green, and blue charts indicate the proportion of *Bactrocera dorsalis*-specific genes in the three GO terms biological process, cellular component, and molecular function, respectively; the pink, light green, and light blue charts represent the proportion of all genes in the three GO terms biological process, cellular component, and molecular function, respectively.

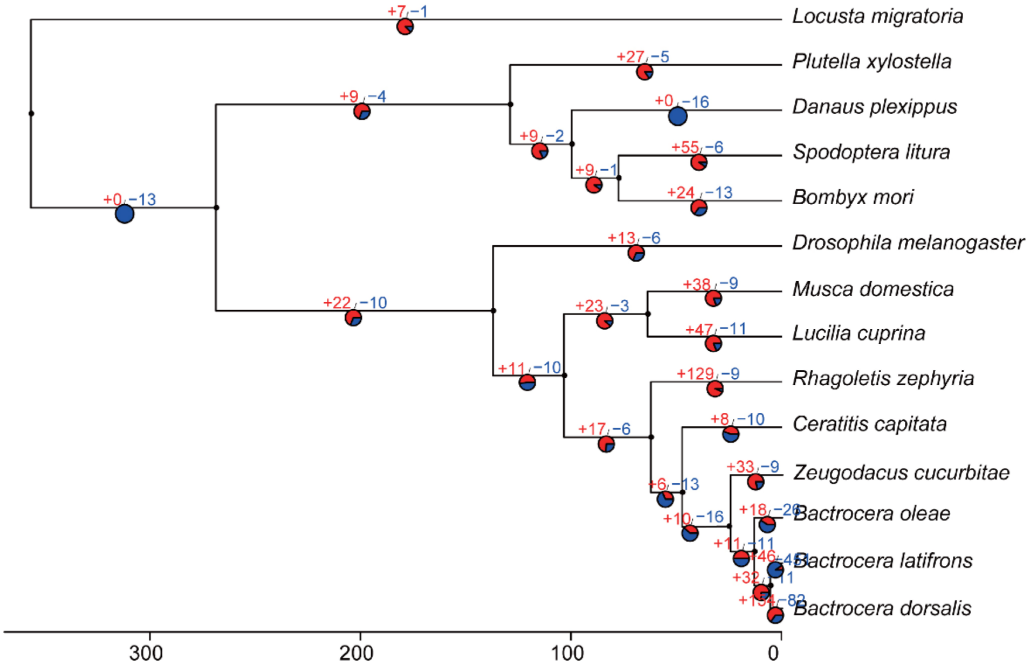

Supplementary Figure 6. Phylogeny of expanded and contracted gene families. Numbers in the tree illustrate the sets of gene-family expansions (+, red) and contractions (–, blue) found for *B. dorsalis*. The pie charts show the proportions of gene family expansions and contractions in a branch.

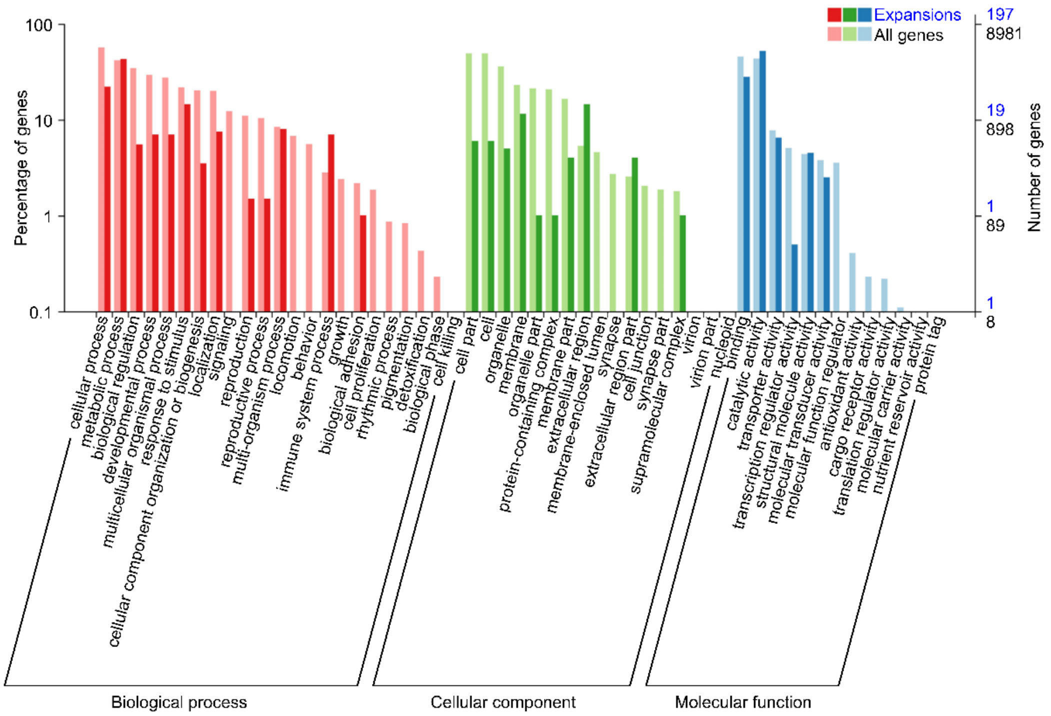

Supplementary Figure 7. All enriched GO terms for the expanded genes in *Bactrocera dorsalis*. The x-axis presents the three GO terms biological process, cellular component, and molecular function. The y-axes in the left and right indicate the percentage of genes and number of genes, respectively. The red, green, and blue charts indicate the proportion of expansion-associated genes in the three GO terms biological process, cellular component, and molecular function, respectively; the pink, light green, and light blue charts represent the proportion of all genes in the three GO terms biological process, cellular component, and molecular function, respectively.

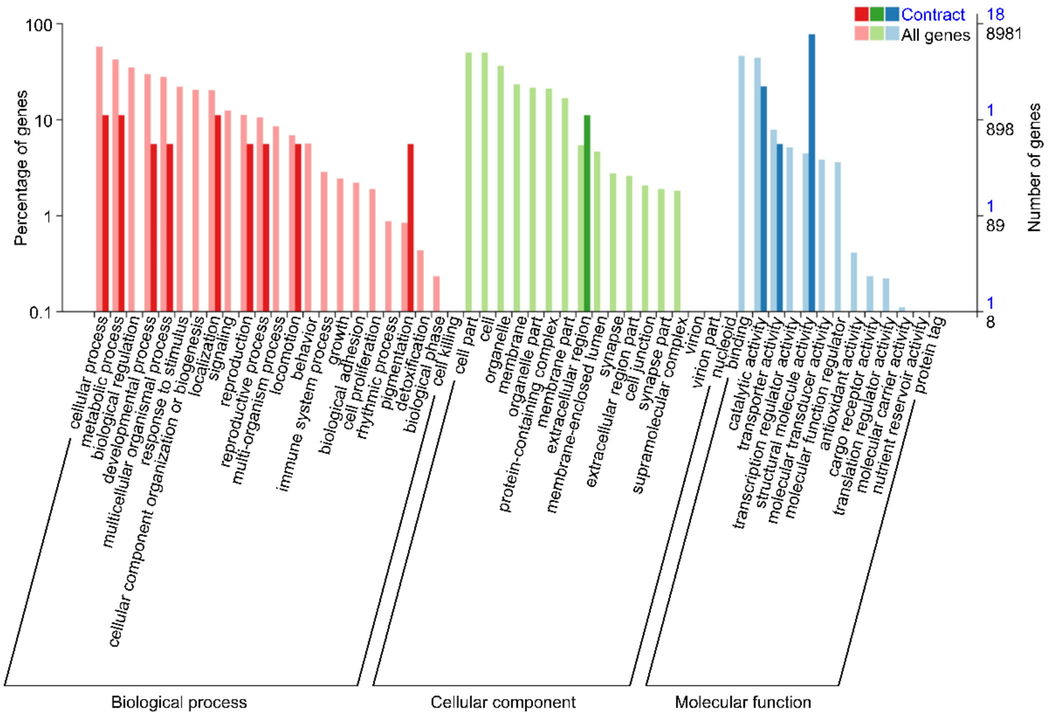

Supplementary Figure 8. All enriched GO terms for the contracted genes in *Bactrocera dorsalis*. The x-axis presents the three GO terms biological process, cellular component, and molecular function. The y-axes in the left and right indicate the percentage of genes and number of genes, respectively. The red, green, and blue charts indicate the proportion of contraction-associated genes in the three GO terms biological process, cellular component, and molecular function, respectively; the pink, light green, and light blue charts represent the proportion of all genes in the three GO terms biological process, cellular component, and molecular function, respectively.
