# Supplementary material for: Chromosome-level genome assembly of Bactrocera dorsalis reveals its adaptation and invasion mechanisms
Source: Commun Biol. 2022 Jan 11;5:25. doi: 10.1038/s42003-021-02966-6 (PMC8752857; doi:10.1038/s42003-021-02966-6)
Supplement: Supplementary file 3 — Description of Additional Supplementary Files [file 42003_2021_2966_MOESM3_ESM.pdf]

Description of Additional Supplementary Files

File name: Supplementary Data 1.
Description: Source data of the main figures in this manuscript.
