# Supplementary material for: Chromosome-level genome assembly of Bactrocera dorsalis reveals its adaptation and invasion mechanisms
Source: Commun Biol. 2022 Jan 11;5:25. doi: 10.1038/s42003-021-02966-6 (PMC8752857; doi:10.1038/s42003-021-02966-6)
Supplement: Supplementary file 5 — Reporting Summary [file 42003_2021_2966_MOESM5_ESM.pdf]

nature portfolio

Corresponding author(s): Shuifang Zhu

Last updated by author(s): Nov 2, 2021

Reporting Summary

Nature Portfolio wishes to improve the reproducibility of the work that we publish. This form provides structure for consistency and transparency in reporting. For further information on Nature Portfolio policies, see our Editorial Policies and the Editorial Policy Checklist.

Statistics

For all statistical analyses, confirm that the following items are present in the figure legend, table legend, main text, or Methods section.

n/a	Confirmed	
☐	☒	The exact sample size (*n*) for each experimental group/condition, given as a discrete number and unit of measurement
☐	☒	A statement on whether measurements were taken from distinct samples or whether the same sample was measured repeatedly
☐	☒	The statistical test(s) used AND whether they are one- or two-sided<br>*Only common tests should be described solely by name; describe more complex techniques in the Methods section.*
☐	☒	A description of all covariates tested
☐	☒	A description of any assumptions or corrections, such as tests of normality and adjustment for multiple comparisons
☐	☒	A full description of the statistical parameters including central tendency (e.g. means) or other basic estimates (e.g. regression coefficient) AND variation (e.g. standard deviation) or associated estimates of uncertainty (e.g. confidence intervals)
☐	☒	For null hypothesis testing, the test statistic (e.g. *F*, *t*, *r*) with confidence intervals, effect sizes, degrees of freedom and *P* value noted<br>*Give P values as exact values whenever suitable.*
☐	☒	For Bayesian analysis, information on the choice of priors and Markov chain Monte Carlo settings
☐	☒	For hierarchical and complex designs, identification of the appropriate level for tests and full reporting of outcomes
☐	☒	Estimates of effect sizes (e.g. Cohen's *d*, Pearson's *r*), indicating how they were calculated

Our web collection on statistics for biologists contains articles on many of the points above.

Software and code

Policy information about availability of computer code

Data collection	No software was used.
Data analysis	Canu v1.5, WTDBG v1.2.8, BWA v0.7.1, LACHESIS, PBjelly2, Genscan, Augustus v2.4, GlimmerHMM v3.0.4, GeneID v1.4, SNAP, GeMoMa v1.3.1, PASA v2.0.2, EVM v1.1.1, tRNAscan-SE v1.3.1, LTR_FINDER v1.05, MITE-Hunter, RepeatScout v1.0.5, PILER-DF v2.4, PASTEClassifier, RepeatMasker v4.0.6, GenBlastA v1.0.4, GeneWise v2.4.1, BLAST2GO, InterProScanv5.8-49.0, Orthofinder v2.3.7, IQ-TREE, MCMCTREE, PAML package v4.9, CAFE v2.2, HMMER 3.0, ClustalW, Evolview v3, TRIMMOMATIC v0.38, HISAT2 v2.1.0, STRINGTIE v2.0, BOWTIE2, RSEM v1.3.1

For manuscripts utilizing custom algorithms or software that are central to the research but not yet described in published literature, software must be made available to editors and reviewers. We strongly encourage code deposition in a community repository (e.g. GitHub). See the Nature Portfolio guidelines for submitting code & software for further information.

Data

Policy information about availability of data

All manuscripts must include a data availability statement. This statement should provide the following information, where applicable:

- Accession codes, unique identifiers, or web links for publicly available datasets
- A description of any restrictions on data availability
- For clinical datasets or third party data, please ensure that the statement adheres to our policy

All sequence data have been deposited at GenBank under the Accession JABETM000000000. Bactrocera dorsalis genome project have been deposited at NCBI GenBank under BioProject Accession PRJNA619226 and BioSample Accession SAMN14492331.

Field-specific reporting

Please select the one below that is the best fit for your research. If you are not sure, read the appropriate sections before making your selection.

☐ Life sciences ☐ Behavioural & social sciences ☒ Ecological, evolutionary & environmental sciences

For a reference copy of the document with all sections, see nature.com/documents/nr-reporting-summary-flat.pdf

Ecological, evolutionary & environmental sciences study design

All studies must disclose on these points even when the disclosure is negative.

Study description	In this study, we aimed to reveal the genome of B. dorsalis at the chromosome-level and find the genetic basis of the invasiveness and rapid adaptation of B. dorsalis by combining various transcriptome data.
Research sample	The B. dorsalis strain was derived from inbred laboratory strains, which were produced through more than 60 generations of sib mating at the Chinese Academy of Inspection and Quarantine (CAIQ), China.
Sampling strategy	To further reduce sequence polymorphisms and achieve a high-quality genome, the samples of B. dorsalis used for de novo sequencing were obtained from one female body using a single mating pair of this strain. Starvation treatment was performed after the samples were obtained.
Data collection	Only the thorax of each sample was retained for genome sequencing. The eggs, larvae, pupae, and mixed B. dorsalis male and female adults were used for transcriptome sequencing using an Illumina HiSeq2500 platform with paired-end libraries for subsequent genome annotation. Data have been submitted to GenBank.
Timing and spatial scale	The B. dorsalis strain used in this study were reared for about five years. We obtained the samples for genome sequencing from Jun. 2017 to Jul. 2017.
Data exclusions	No data were excluded from the analyses
Reproducibility	All attempts to repeat the experiment were successful.
Randomization	We randomly selected the offsprings of B. dorsalis from one female body using a single mating pair of this strain.
Blinding	We only used the samples from laboratory strains for genome sequencing.

Did the study involve field work? ☐ Yes ☒ No

Reporting for specific materials, systems and methods

We require information from authors about some types of materials, experimental systems and methods used in many studies. Here, indicate whether each material, system or method listed is relevant to your study. If you are not sure if a list item applies to your research, read the appropriate section before selecting a response.

Materials & experimental systems

n/a	Involved in the study	
☒	☐	Antibodies
☒	☐	Eukaryotic cell lines
☒	☐	Palaeontology and archaeology
☐	☒	Animals and other organisms
☒	☐	Human research participants
☒	☐	Clinical data
☒	☐	Dual use research of concern

Methods

n/a	Involved in the study	
☒	☐	ChIP-seq
☒	☐	Flow cytometry
☒	☐	MRI-based neuroimaging

Animals and other organisms

Policy information about studies involving animals; ARRIVE guidelines recommended for reporting animal research

Laboratory animals	The B. dorsalis strain was derived from inbred laboratory strains, which were produced through more than 60 generations of sib mating at the Chinese Academy of Inspection and Quarantine (CAIQ), China. To further reduce sequence polymorphisms and achieve a high-quality genome, the samples of B. dorsalis used for de novo sequencing were obtained from one female body using a single mating pair of this strain. Only the thorax of each sample was retained for genome sequencing. The eggs, larvae, pupae, and mixed B. dorsalis male and female adults were used for transcriptome sequencing using an Illumina HiSeq2500 platform with paired-end libraries for subsequent genome annotation.

Wild animals	The study did not involve wild animals.
Field-collected samples	The study did not involve samples collected from the field.
Ethics oversight	No ethical approval was required. The target species in this study is a pest.

Note that full information on the approval of the study protocol must also be provided in the manuscript.
